# Directional selection on cold tolerance does not constrain plastic capacity in a butterfly

**DOI:** 10.1186/1471-2148-12-235

**Published:** 2012-12-05

**Authors:** Kristin Franke, Anneke Dierks, Klaus Fischer

**Affiliations:** 1 Department of Animal Ecology, Zoological Institute and Museum, University of Greifswald, J.-S. Bachstraße 11/12, D-17489, Greifswald, Germany

**Keywords:** Artificial selection, *Bicyclus anynana*, Constraint, Genetic adaptation, Genotype by environment interaction, Phenotypic plasticity, Temperature stress resistance

## Abstract

**Background:**

Organisms may respond to environmental change by means of genetic adaptation, phenotypic plasticity or both, which may result in genotype-environment interactions (G x E) if genotypes differ in their phenotypic response. We here specifically target the latter source of variation (i.e. G x E) by comparing plastic responses among lines of the tropical butterfly *Bicyclus anynana* that had been selected for increased cold tolerance and according controls. Our main aim here was to test the hypothesis that directional selection on cold tolerance will interfere with plastic capacities.

**Results:**

Plastic responses to temperature and feeding treatments were strong, with e.g. higher compared to lower temperatures reducing cold tolerance, longevity, pupal mass, and development time. We report a number of statistically significant genotype-environment interactions (i.e. interactions between selection regime and environmental variables), but most of these were not consistent across treatment groups. We found some evidence though for larger plastic responses to different rearing temperatures in the selection compared to the control lines, while plastic responses to different adult temperatures and feeding treatments were overall very similar across selection regimes.

**Conclusion:**

Our results indicate that plastic capacities are not always constrained by directional selection (on cold tolerance) and therefore genetic changes in trait means, but may operate independently.

## Background

Temperature is considered one of the most important selective agents, and consequently research on temperature stress resistance has attracted much interest over recent decades [1-5]. Temperature-stress resistance refers to an organism’s ability to cope with stressfully high or low temperatures, and is considered a key factor for explaining the distribution and abundance of species [6,7]. Enhanced resistance to temperature stress can be reached by means of phenotypic plasticity, i.e. non-genetic physiological changes as a direct response to environmental variation, or genetic adaptation [5,8]. Genetic variation in temperature stress resistance has been commonly reported in natural systems, with e.g. tropical species showing a higher heat but a lower cold tolerance than temperate-zone species and vice versa [1,3,4,6,7,9-13]. Likewise, high-altitude populations typically show a lower heat but a higher cold tolerance than low-altitude populations [14]. Such geographic variation in fitness-related traits provides strong evidence that these patterns have been shaped by natural selection [14,15]. Several species are also known to respond readily to artificial selection on thermal tolerance traits, providing direct experimental proof for genetic adaptation in temperature stress resistance (e.g. [2,16,17]).

In addition to genetic adaptation, phenotypic plasticity provides a further efficient mechanism to cope with temperature variation [18]. We here define phenotypic plasticity as the set of different phenotypes that may be produced by a single genotype in direct response to different environmental conditions [8]. Plastic responses can be induced during development (developmental plasticity) or in the adult stage (adult acclimation; [5,19,20]), and beneficial effects of a brief exposure to less extreme temperatures are referred to as rapid hardening [1,21,22]. Studies on plastic responses in temperature stress resistance revealed that cooler rearing or adult temperatures increase cold but decrease heat resistance and vice versa, comprising a near universal pattern of putatively adaptive phenotypic plasticity [1,6,23]. For instance, groups of a tropical butterfly reared in a common environment, but being exposed for two days to either 20°C or 27°C in the adult stage, differed subsequently by a factor 2–3 in heat tolerance [23]. Thus, plastic changes in thermal tolerance may be induced within short periods of time, and are highly effective means to deal with temperature variation.

Both genetic variation and phenotypic plasticity may finally interact with one another, resulting in genotype-environment interactions (G x E). We here use G x E to refer to different degrees by which individual genotypes are able to respond to environmental variation [24]. Consequently, a significant G x E demonstrates that some genotypes are more plastic than others [14]. Quantifying such variation is consequently used to explore genetic variation in phenotypic plasticity [25-28]. G x E has been reported for e.g. insect growth and development, fecundity, and immune function [29,30], and is considered to contribute substantially to the maintenance of the genetic variation of traits [31,32]. Although interactions between genotypes and environmental conditions may thus represent a significant source of variation, our respective knowledge is still fairly limited.

Against this background we here investigate G x E in control and selection lines of the tropical nymphalid butterfly *Bicyclus anynana* (Butler, 1879). This species is known to respond readily to temperature manipulations by phenotypic plasticity in temperature stress resistance as well as in an array of other traits [20,23]. Different genotypes of *B. anynana* had been previously produced by applying artificial selection to chill-coma recovery time, yielding highly divergent lines [17]. Chill-coma recovery time is the time an individual needs to regain mobility after cold exposure, and is considered a reliable proxy of climatic cold adaptation [6,23,32,33]. This trait is furthermore known to respond readily to selection as well as to ambient temperatures [2,16,23,34]. We thus exploit the genetic variation generated through artificial selection to investigate whether genotypes substantially varying in cold tolerance respond differentially to environmental manipulations. This explicit focus on G x E contrasts with earlier studies from our group investigating responses and correlated responses to selection [34], effects of selection in the adult stage throughout the life cycle [35], environmental effects on cold tolerance [19], or effects of inbreeding [17]. Specifically we here test the hypotheses that lines selected for increased cold tolerance show reduced plastic responses in cold tolerance (and possibly further traits) compared to control lines. This expectation rests on the assumption that the cold-tolerant lines should benefit less from strong plastic responses in case of critically low temperatures compared to control lines. Further, trait values may already be close to their physiologically determined maximum, constraining further plastic increases.

In order to induce plastic responses we exposed selection and control lines to different rearing and acclimation temperatures as well as to different feeding regimes, and measured the respective effects on cold tolerance and additionally on development and longevity. Some of the concomitant effects reported here are hardly novel, though including environmental manipulations is evidently necessary to investigate G x E. Furthermore this enables us to test a number of additional hypotheses, namely that higher temperatures are predicted to speed up development and to reduce pupal mass, longevity, and cold tolerance [5,20,36,37]. A period of starvation is, based on earlier results, predicted to reduce longevity but to leave cold tolerance unaffected ( [23,36,37]; see Table 1 for specific hypotheses).

**Table 1:** Hypotheses for the main effects of several factors on larval time, pupal time, larval growth rate, pupal mass, chill-coma recovery time, and longevity

**Traits**	**Factors**					
	**Selection regime**	**Inbreeding**	**Rearing temperature**	**Sex**	**Adult temperature**	**Feeding regime**
**Larval time**	No	No	20°C > 27°C	Females > Males	-	-
**Pupal time**	No	No	20°C > 27°C	Males > Females	-	-
**Growth rate**	No	No	27°C > 20°C	Males > Females	-	-
**Pupal mass**	No	No	20°C > 27°C	Females > Males	-	-
**Chill-coma recovery time**	Controls > Selected Lines	No	27°C > 20°C	No	27°C > 20°C	No
**Longevity**	No	No	20°C > 27°C	Females > Males	20°C > 27°C	Control > Starvation

The hypotheses stated rest on prior results mainly obtained from *B. anynana*. Selection for increased cold tolerance is expected to have no effect on any trait other than chill-coma recovery time, as no correlated responses to selection were found in a previous study [34]. No inbreeding effects are expected here due to random mating for 14 generations after full-sib matings and thus prior to scoring trait values. Hypotheses regarding temperature, sex and feeding effects are all straight-forward and rest on previous results [e.g. [5,17,20,23,34,36,37]. ‘-‘: Not tested.

As the initial design of the selection experiment included inbreeding as a factor, we furthermore consider effects of inbreeding in our study. However, based on the fact that the lines were allowed to mate randomly after the different inbreeding levels had been established, no effects of inbreeding are expected anymore (see further below). Selection regime is, based on previous results, also expected to yield no effects on traits other than cold tolerance [34]. We finally include sex as a factor in our analyses, predicting that females show a slower larval but a faster pupal development, a higher pupal mass, and a higher longevity compared to males ( [23], Table 1).

## Methods

### Study organism

*Bicyclus anynana* (Butler 1897; Nymphalidae, Satyrinae) is a tropical fruit-feeding butterfly, ranging from southern Africa to Ethiopia [38]. The species inhabits regions with alternating dry and wet seasons, and shows accordingly two seasonal morphs. During the colder dry season (ca. 18°C, May until November) the species has rather uniform wing patterns and small eyespots, while it exhibits large eyespots and bright bands on both wings in the warmer wet season (ca. 23°C, December until April; [39]). During the dry season reproduction ceases and butterflies do not mate before the onset of the next wet season [29]. Females are relatively monandrous, though multiple mating occurs in the field and in the laboratory [40]. A stock population of *B. anynana* was founded at Greifswald University in 2007 from several hundred individuals derived from a well-established stock population at Leiden University, The Netherlands. The latter was founded in 1988 from over 80 gravid females collected at a single locality in Nkhata Bay, Malawi. To maintain high levels of heterozygosity several hundred adults are reared in each generation [41]. For this experiment animals from the Greifswald stock population were used.

### Experimental design

We here used 12 selection lines that had been previously established at the Department of Animal Ecology, Greifswald University [34,35]. Selection lines for increased cold stress resistance (shorter chill-coma recovery times) and according unselected controls had been derived from three different levels of inbreeding, using a full-sib breeding design: outbred controls (C) having resulted from matings between unrelated butterflies, inbreeding 1 (I1) having resulted from matings between full sibs, and inbreeding 2 (I2) having resulted from matings between full sibs in two consecutive generations. We used ca. 120 full-sib families each for inbreeding levels C, I1, and I2 [34]. At the start of the selection experiment though butterflies were pooled across families within inbreeding levels. Thus, during the course of the selection experiment (10 generations) mating was random within lines. Per inbreeding level, four lines were set up, two for increased cold stress resistance and two unselected controls. This design resulted in a total of 6 selection and 6 control lines (for details see [17,34]). Lines had been kept without selection under standard rearing conditions for 4 generations prior to this experiment. Several hundred butterflies were reared per line in each generation. For the current experiment eggs were collected from all 12 lines. Eggs were thereafter randomly divided among a low (20°C) and a high (27°C) rearing temperature (70 ± 5% relative humidity and photoperiod of L12:D12 throughout). The two temperatures chosen are similar to the ones this species experiences during the wet and dry season in the field, respectively [29]. Larvae were reared in sleeve-like gauze cages, using 10 replicate cages per line and rearing temperature (resulting in a total of 240 cages), and a standard density of 20 (20°C) and 40 (27°C) larvae per cage, respectively. The higher density per cage at 27°C was due to the need for more individuals at this temperature (see below). Care was taken though that developing larvae were never exposed to any food shortage, being fed on young maize plants ad libitum throughout. Resulting pupae were collected daily, weighed one day after pupation, and were then individually transferred to small plastic cups (volume 125 ml). For all animals we scored larval time (from egg-laying until pupation, thus including egg development), pupal time (from pupation until adult eclosion), larval growth rate (ln pupal mass/larval time), and pupal mass (measured on day 2 after pupation).

Following adult eclosion, all butterflies were marked individually and afterwards once again randomly divided among 20°C and 27°C, resulting in four rearing by adult temperature groups per selection line (20-20°C, 20-27°C, 27-20°C, 27-27°C). While the butterflies reared at 27°C were a last time divided among two feeding treatments, being fed with banana (control) or water only (starvation), all animals reared at 20°C were fed with banana ad libitum (to keep the size of the experiment manageable). On day two after eclosion, we tested 24–52 individuals per sex, treatment group and line for chill-coma recovery time, resulting in a total number of 4772 butterflies. Therefore, all butterflies were transferred individually to plastic cups (125 ml), arranged on a tray in a randomized block design, and then exposed to 1°C for 19 h to induce a chill coma. Recovery time (the time until the butterflies were able to stand up) was scored in a climate cell at 20°C. Observations were terminated after 60 minutes. Butterflies that had not recovered by then were given the maximal recovery time of 60 minutes. This applied to < 5% of the butterflies tested.

Thus, we used the same technique for measuring chill-coma recovery time as during selection [35]. This trait is considered a reliable proxy of climatic cold adaptation, is largely independent of the method used to induce a chill coma, and has been used successfully in *B. anynana* before [24]. To afterwards score longevity the butterflies reared at 20°C were returned to their respective adult temperatures and fed ad libitum, while the butterflies reared at 27°C were all transferred to 27°C (due to space limitations) and fed as outlined above until death.

### Statistical analyses

Data on larval time, pupal time, pupal mass, growth rate, chill coma recovery time, and longevity were analyzed with mixed model analyses of variance (ANOVAs), with selection regime (selection vs. control), level of inbreeding (C, I1, I2), rearing temperature (20 vs. 27°C), adult temperature (20 vs. 27°C), adult feeding regime (control vs. starvation, for animals reared at 27°C only), and/or sex as fixed factors. Replicate line was nested within selection regime and inbreeding level, and replicate cage was nested within replicate line, inbreeding level and selection regime. Both latter factors were included as random effects. Minimum adequate models were constructed by sequentially removing non-significant interaction terms. Due to differences in the experimental set-up across rearing temperatures (see above), three different analyses were run for chill-coma recovery time and longevity. For chill-coma recovery time we analyzed all individuals together (note that effects of adult feeding regime were non-significant) and separated by rearing temperature, as different adult feeding regimes were only applied to animals reared at 27°C. For longevity, effects of rearing temperature were analyzed in animals fed ad libitum and kept as adults at 27°C, effects of adult temperature were analyzed in animals reared at 20°C (as only in these different adult temperatures were used throughout), and effects of adult feeding regime were analyzed in animals reared at 27°C (as only in these different adult feeding regimes were applied). Significant differences between inbreeding levels were located using Tukey’s HSD posthoc test. When presenting the statistical results we start with the main effects and continue with interactions, which can be problematic in case of strong interactive effects. Therefore, main effects are interpreted cautiously in case of significant interactions. All statistical tests were performed by using JMP (4.0.0) or Statistica (6.1). Throughout, all means are given ± 1 SE.

## Results

### Larval time

**Figure 1:**
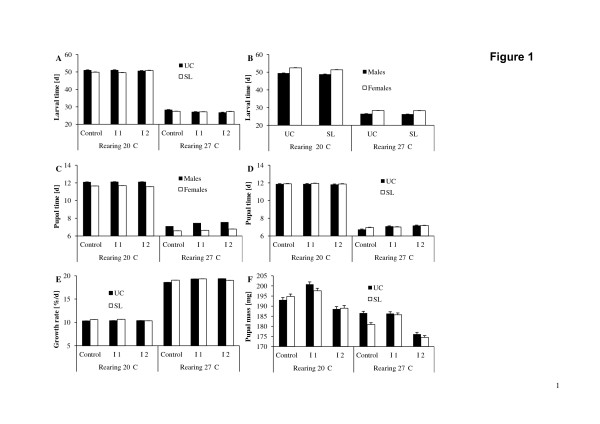
**Variation in life-history traits (means + 1 SE) in relation to various factors in *Bicyclus anynana. *** Larval time in relation to rearing temperature, inbreeding level and selection regime (**A**), and in relation to rearing temperature, selection regime and sex (**B**); pupal time in relation to rearing temperature, inbreeding level and sex (**C**), and in relation to rearing temperature, inbreeding level and selection regime (**D**); larval growth rate in relation to rearing temperature, inbreeding level and selection regime (**E**); and pupal mass in relation to rearing temperature, inbreeding level and selection regime (**F**). UC: unselected control; SL: selection line. Please note that in some cases standard errors are so small that error bars are not visible.

Larval time was significantly affected by rearing temperature, sex, replicate line and replicate cage, but not by selection regime and inbreeding level (Table 2A). Larval time was much longer at the lower compared to the higher rearing temperature (50.6 ± 0.06 d > 27.4 ± 0.05 d; Figure 1A), and was longer in females than in males (40.2 ± 0.06 d > 37.8 ± 0.05 d). The absolute difference in larval time between males and females was smaller at 27°C (26.4 ± 0.06 d vs. 28.3 ± 0.07 d) than at 20°C (49.2 ± 0.09 d vs. 52.0 ± 0.09 d), as indicated by the significant rearing temperature by sex interaction. The sexual difference was particularly small in the unselected controls reared at 27°C (significant selection regime * rearing temperature * sex interaction; Figure 1B). The significant interaction between selection regime and rearing temperature reveals a significantly longer larval time in control than in selection lines at 20°C (51.0 ± 0.09 d > 50.2 ± 0.09 d), which was not the case at 27°C (27.5 ± 0.06 d = 27.3 ± 0.07 d; Tukey HSD). At the higher rearing temperature, larval time was longer in the control compared to the inbred groups (C: 28.0 ± 0.08 d > I1: 27.1 ± 0.08 d = I2: 27.1 ± 0.08 d), while there were no significant differences at 20°C (C: 50.5 ± 0.1 d = I1: 50.3 ± 0.1 d = I2: 50.9 ± 0.1 d; Tukey HSD; significant inbreeding level * rearing temperature interaction). Both above patterns though were not consistent across all groups (significant selection regime * inbreeding level * rearing temperature interaction; Figure 1A).

**Table 2:** Results of nested ANOVAs for larval time (A), pupal time (B), larval growth rate (C) and pupal mass (D)

**A) Larval time**	***DF***	***MS***	***F***	***P***
Selection Regime	1,6	205.6	1.0	0.3453
Inbreeding Level	2,6	87.2	0.4	0.6614
Replicate Line [Sel. Reg. & Inbr.]	6,110	216.0	9.4	**<0.0001**
Repl. Cage [Sel., Inbr. & Repl.]	108,4640	23.5	3.8	**<0.0001**
Rearing Temperature	1,4640	565906.0	90265.2	**<0.0001**
Sex	1,4640	5780.5	922.0	**<0.0001**
Selection Reg. * Inbreeding Level	2,6	236.5	1.2	0.3635
Selection Reg. * Rearing Temp.	1,4640	107.4	17.1	**<0.0001**
Selection Reg. * Sex	1,4640	11.2	1.8	0.1819
Inbreeding Level * Rearing Temp.	2,4640	139.2	22.2	**<0.0001**
Inbreeding Level * Sex	2,4640	14.4	2.3	0.1007
Rearing Temp. * Sex	1,4640	219.6	35.0	**<0.0001**
Sel. Reg. * Inbreed. * Rear. Temp.	2,4640	49.9	8.0	**0.0004**
Sel. Reg. * Rearing Temp. * Sex	1,4640	25.0	25.0	**0.0457**
Error	4640	6.3		
**B) Pupal time**	***DF***	***MS***	***F***	***P***
Selection Regime	1,6	1.4	0.1	0.7627
Inbreeding Level	2,6	9.2	0.6	0.5567
Replicate Line [Sel. Reg. & Inbr.]	6,111	15.6	11.7	**<0.0001**
Repl. Cage [Sel., Inbr. & Repl.]	108,4639	1.4	2.5	**<0.0001**
Rearing Temperature	1,4639	24740.3	45323.1	**<0.0001**
Sex	1,4639	376.4	689.6	**<0.0001**
Selection Reg. * Inbreeding Level	2,6	1.7	0.1	0.8888
Selection Reg. * Rearing Temp.	1,4639	0.2	0.4	0.5196
Selection Reg. * Sex	1,4639	0.8	8.8	0.3708
Inbreeding Level * Rearing Temp.	2,4639	12.8	23.4	**<0.0001**
Inbreeding Level * Sex	2,4639	3.0	5.5	**0.0041**
Rearing Temp. * Sex	1,4639	13.6	24.9	**<0.0001**
Sel. Reg. * Inbreed. * Rear. Temp.	2,3941	3.5	6.3	**0.0018**
Inbreed. * Rear. Temp. * Sex	2,4639	2.2	4.1	**0.0173**
Error	4639	0.5		
**C) Growth rate**	***DF***	***MS***	***F***	***P***
Selection Regime	1,6	5.7	0.1	0.7710
Inbreeding Level	2,6	40.9	0.7	0.5514
Replicate Line [Sel. Reg. & Inbr.]	6,109	68.1	8.2	**<0.0001**
Repl. Cage [Sel., Inbr. & Repl.]	108,4641	8.6	4.3	**<0.0001**
Rearing Temperature	1,4641	79417.9	40343.3	**<0.0001**
Sex	1,4641	154.2	78.3	**<0.0001**
Selection Reg. * Inbreeding Level	2,6	43.2	0.7	0.5353
Selection Reg. * Rearing Temp.	1,4641	2.1	1.1	0.3012
Selection Reg. * Sex	1,4641	0.4	0.2	0.6716
Inbreeding Level * Rearing Temp.	2,4641	33.0	16.8	**<0.0001**
Inbreeding Level * Sex	2,4641	0.6	0.3	0.7479
Rearing Temp. * Sex	1,4641	31.3	15.9	**<0.0001**
Sel. Reg. * Inbreed. * Rear. Temp.	2,4641	8.8	4.5	**0.0115**
Error	4641	2.0		
**D) Pupal mass**	***DF***	***MS***	***F***	***P***
Selection Regime	1,6	2366.2	0.6	0.4880
Inbreeding Level	2,6	38734.6	8.9	**0.0156**
Replicate Line [Sel. Reg. & Inbr.]	6,110	4746.8	3.2	**0.0064**
Repl. Cage [Sel., Inbr. & Repl.]	108,4641	1527.4	3.8	**<0.0001**
Rearing Temperature	1,4641	156622.0	389.2	**<0.0001**
Sex	1,4641	1661991.0	4143.6	**<0.0001**
Selection Reg. * Inbreeding Level	2,6	231.4	0.1	0.9487
Selection Reg. * Rearing Temp.	1,4641	949.7	2.4	0.1239
Selection Reg. * Sex	1,4641	251.4	0.6	0.4286
Inbreeding Level * Rearing Temp.	2,4641	1285.1	3.2	**0.0407**
Inbreeding Level * Sex	2,4641	1253.2	3.1	**0.0441**
Rearing Temp. * Sex	1,4641	295.4	0.7	0.3908
Sel. Reg. * Inbreed. * Rear. Temp.	2,4641	2182.8	5.4	**0.0044**
Error	4641	400.5		

Results for the effects of selection regime (selection vs. control), inbreeding level (C, I1, I2), replicate line, replicate cage, rearing temperature (20 vs. 27°C), and sex on larval time (A), pupal time (B), larval growth rate (C), and pupal mass (D) in *Bicyclus anynana*. Replicate line was nested within selection regime and inbreeding level, and replicate cage was nested within replicate line, inbreeding level and selection regime. Both latter factors were included as random effects, while all others were considered fixed effects. Minimum adequate models were constructed by sequentially removing non-significant interaction terms. Significant *P*-values are given in bold.

### Pupal time

Pupal time was significantly influenced by rearing temperature, sex, replicate cage and replicate line, but not by selection regime and inbreeding level (Table 2B). Pupal time was longer at the lower compared to the higher rearing temperature (11.9 ± 0.02 d > 7.0 ± 0.01 d), and was longer in males than in females (9.7 ± 0.02 d > 9.2 ± 0.02 d). A significant rearing temperature by sex interaction indicates that sexual differences were slightly more pronounced at 27°C (7.4 ± 0.02 d > 6.7 ± 0.02 d) than at 20°C (12.1 ± 0.03 d > 11.6 ± 0.03 d; Figure 1C). At the higher rearing temperature, pupal time increased with increasing inbreeding level (C: 6.8 ± 0.02 d < I1: 7.1 ± 0.02 d < I2: 7.2 ± 0.02 d), while at the lower rearing temperature inbreeding level had no significant effect (C: 11.9 ± 0.03 d = I1: 11.9 ± 0.03 d = I2: 11.8 ± 0.03 d; Tukey HSD; significant inbreeding level * rearing temperature interaction). Furthermore, inbreeding effects on pupal time were more pronounced in males (C: 9.6 ± 0.03 d < I1: 9.8 ± 0.03 d = I2: 9.8 ± 0.03 d) than in females (C: 9.1 ± 0.03 d = I1: 9.2 ± 0.03 d = I2: 9.2 ± 0.03 d; Tukey HSD; significant inbreeding level * sex interaction). Consequently, inbreeding effects were largely restricted to males reared at 27°C (significant inbreeding level * rearing temperature * sex interaction; Figure 1C). The significant interaction between selection regime, inbreeding level and rearing temperature indicates that differences between selection and control lines were basically limited to the outbred control group when reared at 27°C (Figure 1D).

### Growth rate

Growth rate was also significantly affected by rearing temperature, sex, replicate line and replicate cage, but not by selection regime and inbreeding level (Table 2C). Growth rate was higher at the higher compared to the lower rearing temperature (19.1 ± 0.03%/d > 10.4 ± 0.03%/d), and was higher in males than in females (14.9 ± 0.03%/d > 14.6 ± 0.03%/d). The significant interaction between rearing temperature and sex indicates a larger sexual difference in growth rates at 27°C (males: 19.4 ± 0.04%/d > females: 18.8 ± 0.04%/d) than at 20°C (males: 10.5 ± 0.05%/d > females: 10.3 ± 0.05%/d). Effects of inbreeding were found at the higher rearing temperature only (C: 18.8 ± 0.04%/d < I1: 19.3 ± 0.04%/d = I2: 19.2 ± 0.05%/d), but not at the lower one (C: 10.4 ± 0.06%/d = I1: 10.5 ± 0.06%/d = I2: 10.3 ± 0.06%/d; Tukey HSD, significant inbreeding level * rearing temperature interaction). Moreover, clear evidence for effects of inbreeding on growth rates was restricted to the unselected control lines reared at 27°C (significant selection regime * inbreeding level * rearing temperature interaction; Figure 1E).

### Pupal mass

Pupal mass differed significantly between inbreeding levels, rearing temperatures, sexes, replicate lines and replicate cages, but not in relation to selection regime (Table 2D). Pupal mass was highest in the inbreeding 1 group, followed by the outbred control and finally the inbreeding 2 group (I1: 192.7 ± 1.72 mg > C: 188.9 ± 1.74 mg > I2: 182.1 ± 1.85 mg; Tukey HSD). The exact patterns though differed among the higher (I1: 186.0 ± 0.63 mg = C: 183.9 ± 0.64 mg > I2: 175.4 ± 0.65 mg) and the lower rearing temperature (I1: 199.3 ± 0.83 mg > C: 193.9 ± 0.85 mg > I2: 188.7 ± 0.91 mg; Tukey HSD; significant inbreeding level * rearing temperature interaction), and also among the sexes (males: I1: 173.1 ± 0.73 mg > C: 168.0 ± 0.73 mg > I2: 163.0 ± 0.77 mg; females: I1: 212.2 ± 0.74 mg = C: 209.7 ± 0.74 mg > I2: 201.2 ± 0.78 mg; significant inbreeding level * sex interaction). Effects of selection regime were absent except for the outbred controls reared at 27°C, where pupal mass was higher in the unselected controls (significant selection regime * inbreeding level * rearing temperature interaction; Figure 1F). Pupal mass was overall higher at 20°C than at 27°C (194.0 ± 0.50 mg > 181.8 ± 0.37 mg), and was higher in females than in males (207.7 ± 0.44 mg > 168.0 ± 0.43 mg).

### Chill-coma recovery time

**Figure 2:**
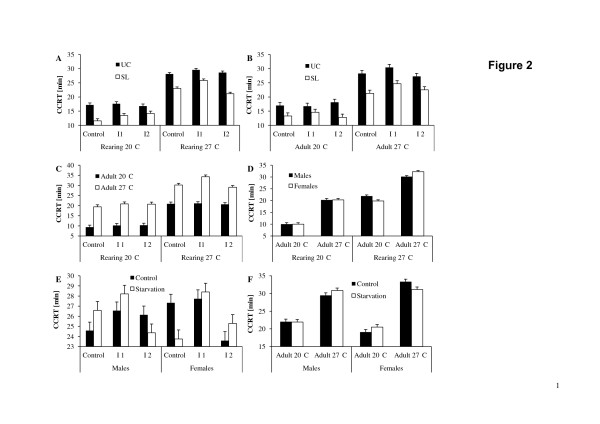
**Variation in chill-coma recovery time (means + 1 SE) in *Bicyclus anynana. *** Chill-coma recovery time in relation to rearing temperature, inbreeding level and selection regime (**A**), adult temperature, inbreeding level and selection regime (**B**), rearing temperature, inbreeding level and adult temperature (**C**), rearing temperature, adult temperature and sex (**D**), sex, inbreeding level and adult feeding regime (**E**), and sex, adult temperature and adult feeding regime (**F**). Graphs A-D refer to all animals, while graphs E-F refer only to the animals reared at 27°C. UC: unselected control; SL: selection line; CCRT: Chill-coma-recovery-time.

Chill-coma recovery time was significantly affected by selection regime, rearing temperature, adult temperature, and replicate line, but not by inbreeding level and sex (Table 3A). Animals from the selection lines recovered faster than those from the control lines (18.2 ± 0.73 min > 23.0 ± 0.70 min). Furthermore, animals reared at 20°C recovered faster than those reared at 27°C (15.2 ± 0.31 min < 26.0 ± 0.23 min), as was the case for animals acclimated to 20°C compared to 27°C (15.4 ± 0.27 min < 25.8 ± 0.27 min). The significant interaction between selection regime and adult temperature reveals that the difference between selection lines and unselected controls was larger at 27°C (22.9 ± 0.38 min versus 28.7 ± 0.37 min) than at 20°C (13.6 ± 0.38 min versus 17.3 ± 0.37 min). Evidence for effects of inbreeding on chill-coma recovery time were restricted to animals reared at 27°C (C: 25.5 ± 0.39 min = I2: 24.8 ± 0.39 min < I1: 27.7 ± 0.39 min), while no effects of inbreeding were found in animals reared at 20°C (C: 14.4 ± 0.52 min = I2: 15.6 ± 0.55 min = I1: 15.5 ± 0.52 min, Tukey HSD; significant inbreeding level * rearing temperature interaction). More specifically, the selection lines reared at 27°C caused the above pattern, while the unselected control lines showed neither at 20°C nor at 27°C a significant response to inbreeding, and while the selection lines reared at 20°C showed an increase in chill-coma recovery time with increasing inbreeding level (significant selection regime * inbreeding level * rearing temperature interaction; Figure 2A).

**Table 3:** Results of nested ANOVAs for chill-coma recovery time

**A) Chill-coma recovery time**	***DF***	***MS***	***F***	***P***
Selection Regime	1,6	24368.4	22.0	**0.0032**
Inbreeding Level	2,6	1183.4	1.1	0.4005
Replicate Line [Sel. & Inbr.]	6,4738	1204.3	7.7	**<0.0001**
Rearing Temperature	1,4738	127708.0	815.0	**<0.0001**
Adult Temperature	1,4738	115643.0	738.0	**<0.0001**
Sex	1,4738	<0.1	<0.1	0.9926
Selection Reg. * Inbreeding Level	2,6	192.6	0.2	0.8448
Selection Reg.* Rearing Temp.	1,4738	480.4	3.1	0.0800
Selection Reg. * Adult Temp.	1,4738	1327.2	8.5	**0.0036**
Selection Reg. * Sex	1,4738	21.2	0.1	0.7131
Inbreeding Level * Rearing Temp.	2,4738	797.1	5.1	**0.0062**
Inbreeding Level * Adult Temp.	2,4738	692.4	4.4	**0.0121**
Inbreeding Level * Sex	2,4738	254.0	1.6	0.1979
Rearing Temp. * Adult Temp.	1,4738	1.2	<0.1	0.9298
Rearing Temp. * Sex	1,4738	1.8	<0.1	0.9140
Adult Temp. * Sex	1,4738	1384.2	8.8	**0.0030**
Sel. Reg. * Inbreed. * Rear. Temp.	2,4738	910.95	5.8	**0.0030**
Sel. Reg. * Inbreed. * Adult Temp.	2,4738	527.1	3.4	**0.0347**
Inbreed. * Rear. Temp. * Adult Temp.	2,4738	504.9	3.2	**0.0400**
Rear. Temp. * Adult Temp. * Sex	1,4738	1121.7	7.2	**0.0075**
Error	4738	156.7		
**B) Animals reared at 20°C**	***DF***	***MS***	***F***	***P***
Selection Regime	1,6	6782.9	18.8	**0.0049**
Inbreeding Level	2,6	213.8	0.6	0.5829
Replicate Line [Sel. & Inbr.]	6,1668	362.6	4.1	**0.0004**
Adult Temperature	1,1668	44483.1	501.1	**<0.0001**
Sex	1,1668	1.3	<0.1	0.9046
Selection Reg. * Adult Temp.	1,1668	1458.2	16.4	**<0.0001**
Error	1668	88.8		
**C) Animals reared at 27°C**	***DF***	***MS***	***F***	***P***
Selection Regime	1,6	23033.8	15.3	**0.0079**
Inbreeding Level	2,6	2340.1	1.5	0.2859
Replicate Line [Sel. & Inbreed.]	6,3059	1506.7	7.9	**<0.0001**
Adult Temperature	1,3059	81814.6	427.7	**<0.0001**
Adult Feeding Regime	1,3059	5.6	<0.1	0.8644
Sex	1,3059	0.4	<0.1	0.9620
Selection Reg. * Inbreeding Level	2,6	959.2	0.6	0.5611
Selection Reg. * Adult Temp.	1,3059	321.2	1.7	0.1952
Selection Reg. * Adult Feed.	1,3059	66.0	0.3	0.5570
Selection Reg. * Sex	1,3059	38.0	0.2	0.6557
Inbreeding Level * Adult Temp.	2,305	1643.0	8.6	**0.0002**
Inbreeding Level * Adult Feed.	2,3059	211.2	1.1	0.3317
Inbreeding Level * Sex	2,3059	145.2	0.8	0.4682
Adult Temp. * Adult Feed.	1,3059	231.8	1.2	0.2711
Adult Temp. * Sex	1,3059	3459.7	18.1	**<0.0001**
Adult Feed. * Sex	1,3059	174.4	0.9	0.3397
Sel. Reg. * Inbreed. * Adult Temp.	2,3059	961.3	5.0	**0.0066**
Inbreed. * Adult Feed. * Sex	2,3059	1243.4	6.5	**0.0015**
Adult Temp. * Adult Feed. * Sex	1,3059	1362.7	7.1	**0.0076**
Error	3059	191.3		

Results for the effects of selection regime (selection vs. control), inbreeding level (C, I1, I2), replicate line, rearing temperature (20 vs. 27°C), adult temperature (20 vs. 27°C), and sex on chill-coma recovery time in *Bicyclus anynana* (A). In Tables B (animals reared at 20°C) and C (animals reared at 27°C) data were analyzed separately for each rearing temperature, as different feeding treatments were employed in animals reared at 27°C only. Consequently, the factor rearing temperature was dropped from both analyses, and the factor adult feeding regime (control vs. starvation) was added to Table C. For details see Material and Methods. Replicate line (random factor) was nested within selection regime and inbreeding level throughout. Minimum adequate models were constructed by sequentially removing non-significant interaction terms. Significant *P*-values are given in bold.

Likewise, inbreeding effects were only detectable in animals acclimated to 27°C (C: 24.8 ± 0.46 min = I2: 24.9 ± 0.48 min < I1: 27.6 ± 0.46 min), but not in those acclimated to 20°C (C: 15.1 ± 0.46 min = I2: 15.5 ± 0.48 min = I1: 15.7 ± 0.46 min; Tukey HSD; significant inbreeding level * adult temperature interaction). The lack of a comparable response in the butterflies acclimated to 20°C is caused by the unselected controls, showing an increase in recovery time with increasing inbreeding level (significant selection regime * inbreeding level * adult temperature interaction; Figure 2B). Overall, inbreeding effects were thus only detectable in the animals reared at and acclimated to 27°C, but not in any other rearing by adult temperature group (significant inbreeding level * rearing temperature * adult temperature interaction; Figure 2C). The significant adult temperature by sex interaction indicates that males tended to show longer recovery times than females at the adult temperature of 20°C (16.0 ± 0.38 min vs. 14.9 ± 0.38 min), but shorter ones than females at 27°C (25.2 ± 0.37 min vs. 26.3 ± 0.39 min). This pattern was restricted to animals reared at 27°C though, while sexual differences were absent in animals reared at 20°C (significant rearing temperature * adult temperature * sex interaction; Figure 2D).

When analyzing the animals reared at 20°C separately, selection regime, adult temperature and replicate line significantly affected chill-coma recovery time, but not inbreeding level and sex (Table 3B). Butterflies from the selection lines recovered quicker compared to unselected control butterflies (13.2 ± 0.70 min < 17.2 ± 0.66 min), and adults kept at 20°C recovered much faster than those kept at 27°C (10.1 ± 0.32 min < 20.3 ± 0.33 min). All patterns are thus in full agreement with the above analysis. The interaction between selection regime and adult temperature indicates that differences in chill-coma recovery time between selection and control lines were more pronounced at the higher (17.4 ± 0.47 min vs. 23.3 ± 0.45 min) compared to the lower adult temperature (9.0 ± 0.47 min vs. 11.1 ± 0.45 min).

In the animals reared at 27°C, chill-coma recovery time was significantly affected by selection regime, adult temperature, and replicate line, but not by adult feeding regime, sex and inbreeding level (Table 3C). Butterflies from the selection lines had shorter recovery times than those from the unselected control lines (23.3 ± 0.99 min vs. 28.8 ± 0.99 min), and individuals acclimated to the lower temperature recovered faster than those acclimated to the higher temperature (20.9 ± 0.36 min < 31.2 ± 0.35 min). The significant interactions found here basically reflect the patterns described above, and are thus not described here except for both interactions involving feeding regime. The response to adult feeding regime varied largely among inbreeding levels and sexes, with feeding regime having positive to negative effects (significant inbreeding level * adult feeding regime * sex interaction; Figure 2E). Furthermore, food stress had generally either little or positive effects on chill-coma recovery times in the adult temperature by sex groups, while it slightly decreased cold stress resistance in the females acclimated to 27°C (significant adult temperature * adult feeding regime * sex interaction; Figure 2F).

### Longevity

**Figure 3:**
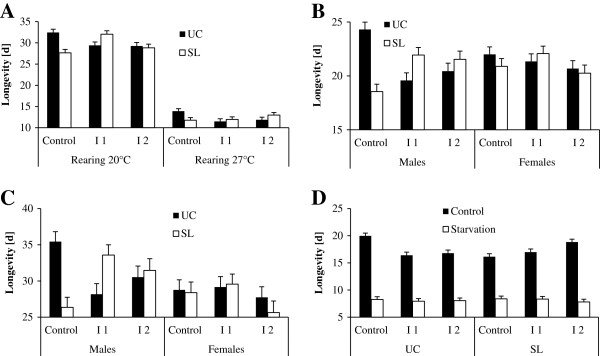
**Variation in longevity (means + 1 SE) in *Bicyclus anynana. *** Longevity in relation to rearing temperature, inbreeding level and selection regime (**A**), sex, inbreeding level and selection regime (**B**), sex, inbreeding level and selection regime (**C**), and selection regime, inbreeding level and adult feeding treatment (**D**). Graphs A-B refer to animals that were fed ad libitum and kept at 27°C as adults, graph C to the animals reared at 20°C, and graph D to those reared at 27°C. UC: unselected control; SL: selection line.

In the analysis addressing effects of rearing temperature, longevity was significantly affected by rearing temperature and replicate line, but not by selection regime, inbreeding level, and sex (Table 4A). Animals reared at 20°C lived longer compared to animals reared at 27°C (29.9 ± 0.33 d > 12.3 ± 0.25 d). The significant selection regime by inbreeding level interaction indicates that longevity tended to be longer in the unselected controls than in the selection lines in the outbred controls (23.2 ± 0.78 d vs. 19.7 ± 0.81 d), but shorter in inbreeding group 1 (20.5 ± 0.81 d vs. 22.0 ± 0.79 d), while being very similar in inbreeding group 2 (20.6 ± 0.84 d vs. 20.9 ± 0.87 d). These patterns, however, were largely restricted to animals reared at 20°C and to males (significant selection regime * inbreeding level * rearing temperature interaction and significant selection regime * inbreeding level * sex interaction; Figure 3A and 3B). Consequently, significant differences between selection and control lines were exclusively found in outbred males reared at 20°C (35.5 ± 1.1 d vs. 26.3 ± 1.1 d; significant four-way interaction). When being reared at 20°C, longevity was longest in inbreeding 1 group (C: 30.0 ± 0.56; I1: 30.7 ±0.56 d; I2: 29.0 ± 0.61 d), while it was shortest in this group when being reared at 27°C (C: 12.8 ± 0.42; I1: 11.7 ± 0.42 d; I2; 12.4 ± 0.43 d; significant inbreeding level * rearing temperature interaction). Sex differences were only found in animals reared at 20°C (males: 31.1 ± 0.47 d > females: 28.8 ± 0.47 d), while there was no significant sex difference in animals reared at 27°C (males: 11.0 ± 0.35 d = females: 13.7 ± 0.35 d; Tukey HSD; significant rearing temperature * sex interaction).

**Table 4:** Results of nested ANOVAs for longevity

**A) Longevity**	***DF***	***MS***	***F***	***P***
Selection Regime	1,6	234.0	0.6	0.4616
Replicate Line [Sel. & Inbr.]	6,3744	404.9	2.8	**0.0109**
Inbreeding Level	2,6	146.3	0.4	0.6967
Rearing Temperature	1,3744	264262.0	1806.6	**<0.0001**
Sex	1,3744	20.7	0.14	0.7070
Selection Reg. * Inbreeding Level	2,6	2020.7	5.3	**0.0440**
Selection Reg. * Rearing Temp.	1,3744	93.4	0.6	0.4242
Selection Reg. * Sex	1,3744	52.2	0.4	0.5505
Inbreeding Level * Rearing Temp.	2,3744	440.1	3.0	**0.0495**
Inbreeding Level * Sex	2,3744	153.7	1.0	0.3498
Rearing Temp. * Sex	1,3744	5324.0	36.4	**<0.0001**
Sel. Reg. * Inbreed. * Rear. Temp.	2,3744	472.0	3.2	**0.0398**
Sel. Reg. * Inbreed. * Sex	2,3744	940.0	6.4	**0.0016**
Sel. Reg. * Rearing Temp. * Sex	1,3744	1.5	<0.1	0.9202
Inbreed. * Rear. Temp. * Sex	2,3744	178.4	1.2	0.2954
Four-way Interaction	2,3744	932.1	6.4	**0.0017**
Error	3744	146.3		
**B) Animals reared at 20°C**	***DF***	***MS***	***F***	***P***
Selection Regime	1,6	287.5	0.3	0.6267
Inbreeding Level	2,6	174.0	0.2	0.8570
Replicate Line [Sel. Reg. & Inbr.]	6,1312	1106.3	4.8	**<0.0001**
Adult Temperature	1,1312	34430.6	148.6	**<0.0001**
Sex	1,1312	2568.9	11.1	**0.0009**
Selection Reg. * Inbreeding Level	2.6	1712.1	1.6	0.2864
Selection Reg. * Adult Temp.	1,1312	1726.0	7.5	**0.0064**
Selection Reg. * Sex	1,1312	7.3	<0.1	0.8589
Inbreeding Level * Adult Temp.	2,1312	3034.3	13.5	**<0.0001**
Inbreeding Level * Sex	2,1312	131.9	0.6	0.5659
Adult Temperature * Sex	1,1312	2762.2	12.6	**0.0004**
Sel. Reg. * Inbreed. * Sex	2,1312	1395.7	6.0	**0.0025**
Error	1312	231.6		
**C) Animals reared at 27°C**	***DF***	***MS***	***F***	***P***
Selection Regime	1,6	38.0	0.1	0.7603
Inbreeding Level	2,6	142.8	0.4	0.6974
Replicate Line [Sel. Reg. & Inbr.]	6,2394	37.	7.0	**<0.0001**
Adult Temperature	1,2394	1177.7	21.8	**<0.0001**
Adult Feeding Regime	1,2394	52827.7	977.2	**<0.0001**
Sex	1,2394	3684.1	68.1	**<0.0001**
Selection Reg. * Inbreeding Level	2,6	475.6	1.3	0.3451
Selection Reg. * Adult Temp.	1,2394	104.6	1.9	0.1644
Selection Reg. * Adult Feed.	1,2394	49.3	0.9	0.3399
Selection Reg. * Sex	1,2394	15.4	0.3	0.5936
Inbreeding Level * Adult Temp.	2,2394	37.1	0.7	0.5033
Inbreeding Level * Adult Feed.	2,2394	112.8	2.1	0.1244
Inbreeding Level * Sex	2,2394	137.9	2.6	0.0782
Adult Temp. * Adult Feed.	1,2394	451.8	8.4	**0.0039**
Adult Temp. * Sex	1,2394	114.8	2.1	0.1451
Adult Feed. * Sex	1,2394	1481.2	27.4	**<0.0001**
Sel. Reg. * Inbreed. * Adult Temp.	2,2394	17.4	0.3	0.7248
Sel. Reg. * Inbreed. *Adult Feed.	2,2394	532.5	9.9	**<0.0001**
Sel. Reg. * Inbreed. * Sex	22394	0.7	<0.1	0.9879
Sel. Reg. * Adult Temp. * Sex	2,2394	1.7	<0.1	0.8610
Sel. Reg. * Adult Temp. * AdFeed.	1,2394	128.8	2.4	0.1229
Sel. Reg. * Adult Feed. * Sex	1,2394	0.7	<0.1	0.9110
Inbreed. * Adult Temp. * AdFeed.	2,2394	14.4	0.3	0.7659
Inbreed. * Adult Temp. * Sex	2,2394	161.9	3.0	0.0502
Inbreed. * Adult Feed. * Sex	2,2394	164.2	3.0	**0.0482**
Adult Temp. * Adult Feed. * Sex	1,2394	87.1	1.6	0.2044
Inbreed. * AdT * AdF * Sex	2,2394	222.8	4.1	**0.0163**
Error	2394	54.0		

Results for the effects of selection regime (selection vs. control), inbreeding level (C, I1, I2), replicate line, replicate cage, rearing temperature (20 vs. 27°C), and sex on longevity in *Bicyclus anynana* (A). In Tables B (animals reared at 20°C) and C (animals reared at 27°C) data were analyzed separately for each rearing temperature, as different feeding treatments were employed in animals reared at 27°C only, and as different adult temperatures were employed in animals reared at 27°C only temporarily. Consequently, the analysis in A was restricted to the animals fed ad libitum and kept as adults at 27°C, to mainly explore effects of rearing temperature. The factor rearing temperature was replaced by the factor adult temperature in analyses B and C, and the factor adult feeding regime (control vs. starvation) was added to Table C. For details see Material and Methods. Replicate line (random factor) was nested within selection regime and inbreeding level throughout. Minimum adequate models were constructed by sequentially removing non-significant interaction terms. Significant *P*-values are given in bold.

When considering the animals reared at 20°C separately, adult temperature, sex, and replicate line significantly affected longevity, but not selection regime and inbreeding level (Table 4B). Adults acclimated to 20°C lived longer than adults acclimated to 27°C (34.7 ± 0.57 d > 24.4 ± 0.62 d), and males lived longer than females (30.9 ± 0.60 d > 28.1 ± 0.60 d). The sex difference was restricted to the adult temperature of 27°C though (males: 27.3 ± 0.87 d > females: 21.5 ± 0.88 d), while there was no significant difference at 20°C (males: 34.6 ± 0.82 d = females: 34.7 ± 0.80 d; Tukey HSD; significant adult temperature * sex interaction). The significant interaction between selection regime and adult temperature indicates that longevity was longer in unselected controls compared to selection lines at the lower adult temperature (36.2 ± 0.80 d vs. 33.1 ± 0.82 d), but vice versa at the higher adult temperature (23.8 ± 0.86 d vs. 24.9 ± 0.88 d). Longevity decreased with increasing inbreeding level when animals were acclimated to 20°C (C: 37.2 ± 0.98 d = I1: 35.8 ± 0.94 d > I2: 31.0 ± 1.05 d), but tended to increase with increasing inbreeding level when animals were acclimated to 27°C (C: 22.2 ± 1.0 d = I1: 24.4 ± 1.06 d = I2: 26.6 ± 1.13 d; Tukey HSD; significant inbreeding level * adult temperature interaction). Finally, males of the outbred control and the inbreeding 1 group showed quite substantial though opposing differences between selection lines and unselected controls, while differences where much smaller in other groups (significant selection regime * inbreeding level * sex interaction; Figure 3C).

In the animals reared at 27°C, longevity was significantly affected by adult temperature, adult feeding regime, sex and replicate line, but not by selection regime and inbreeding level (Table 4C). Adults acclimated to 20°C lived longer than adults acclimated to 27°C (13.5 ± 0.21 d > 12.1 ± 0.21 d), control animals lived longer compared to food-stressed animals (17.5 ± 0.22 d > 8.1 ± 0.20 d), and females lived longer than males (14.1 ± 0.21 d > 11.6 ± 0.21 d). Effects of adult temperature were more pronounced in control (20°C: 18.7 ± 0.32 d vs. 27°C: 16.4 ± 0.31 d) than in food-stressed animals (20°C: 8.4 ± 0.29 d vs. 27°C: 7.8 ± 0.28 d; significant adult temperature * adult feeding regime interaction). Sex differences, in contrast, were larger in food-stressed (female: 10.2 ± 0.29 d vs. male: 6.1 ± 0.28 d) than in control animals (female: 18.0 ± 0.32 d vs. male: 17.1 ± 0.31 d; significant adult feeding regime * sex interaction). While in food-stressed animals longevity was generally very similar across selection regimes and inbreeding levels, the feeding control groups showed divergent patterns between unselected controls and selection lines (Figure 3D). While in the latter groups longevity increased with increasing inbreeding level in the selection lines (C: 16.1 ± 0.56 d = I1: 16.9 ± 0.56 d < I2: 18.8 ± 0.54 d), it decreased with increasing inbreeding level in the unselected control lines (C: 20.2 ± 0.51 d > I1: 16.4 ± 0.56 d = I2: 16.8 ± 0.56 d; significant selection regime * inbreeding level * adult feeding regime interaction). The significant inbreeding level * adult feeding regime * sex interaction shows that inbreeding effects were only present in food control males, in which individuals from inbreeding level 1 lived shortest (I1: 15.5 ± 0.56 d < I2: 17.0 ± 0.55 d = C: 18.7 ± 0.53 d). Variation between inbreeding groups though was restricted to food control males kept at 20°C (I1: 16.1 ± 0.79 d; I2: 18.1 ± 0.78 d; C: 21.6 ± 0.76 d; significant inbreeding level * adult temperature * adult feeding regime * sex interaction).

## Discussion

### Variation in developmental traits

As predicted (Table 1), selection regime neither affected larval time, pupal time, larval growth rate or pupal mass, thus suggesting that genetic variation in adult cold tolerance operates largely independent of these developmental traits in *B. anynana* (see [17]; cf. [16,42]). However, selection regime was involved in a total of six interactions with other factors (larval time: 3; pupal time: 1; growth rate: 1; pupal mass: 1; Table 2). The patterns revealed through these interactions though do not fundamentally change the conclusion drawn above, with perhaps the following exception. Larval development time was slightly longer in control than in selection lines at the lower rearing temperature only. This may indicate that the lines selected for increased adult cold tolerance may be slightly better adapted to deal with developmental temperatures below the optimal range. Note though that this pattern was not consistent across all treatment groups (significant 3-way interaction). All other interactions indicated inconsistent, group-specific responses and did therefore not reveal any straightforward effects of selection regime on developmental traits.

Likewise, inbreeding yielded only marginal effects, significantly affecting pupal mass only. Pupal mass was highest in inbreeding group 1, intermediate in the outbred control, and lowest in inbreeding group 2. Directly after the full-sib matings, in contrast, larval development time, larval growth rate, and pupal mass showed inbreeding depression [17]. The fact that much of these initial differences disappeared indicates fitness rebounds caused by subsequent random mating (for 14 generations: 10 during the selection experiments and 4 afterwards) as was expected [17]. Additive theory would predict a complete rebound under such circumstances [43,44], such that the pattern found for pupal mass may reflect a chance effect caused by random genetic drift rather than inbreeding depression. Note in this context that the 11 interactions in which inbreeding was involved in also indicated erratic variation rather than any systematic patterns.

In contrast to the above results, sexual differences and variation induced by different rearing temperatures resemble predicted and well-known patterns for *B. anynana* and beyond [45,46]. Specifically, lower temperatures resulted in increased development time and pupal mass, but in decreased larval growth rates. This is consistent with the temperature-size rule and the in general temperature-dependent growth of ectotherms [46-48]. Males compared to females showed shorter larval but longer pupal times (though with the latter not affecting earlier male emergence; protandry selection), higher larval growth rate, and lower pupal mass (fecundity selection in females). Sex differences were more pronounced at 20°C (larval time) or at 27°C (pupal time, larval growth rates). While the former has been reported previously and likely reflects larger absolute differences owing to the overall longer development time at lower temperatures [49], we have no explanation for the latter.

### Variation in chill-coma recovery time

Cold tolerance was strongly affected by selection regime, with recovery times being overall by 21% shorter in the selection than in the control lines. Directly after the course of selection the difference between selection regimes was 29% [17], indicating an ongoing convergence towards control line levels. This suggests that the selected genes and alleles had not yet become fixed in the selection lines, and furthermore a lack of selection for maintaining increased cold tolerance under laboratory conditions. Interestingly, the differences between selection and control lines tended to be more pronounced when animals had been reared at 20°C (23%) as compared to 27°C (18%; Table 3A). This suggests that the selection lines might show a somewhat stronger response to rearing temperature (by 43% shorter chill-coma recovery time at 20°C compared to 27°C) than the control lines (by 40% shorter).

Regarding interactive effects, selection regime was involved in a total of five significant interactions. While three of these did not reveal any conclusive patterns regarding the role of selection regime, the remaining two interactions suggest that the differences between selection and control lines were larger at the higher as compared to the lower adult temperature (Table 3 A and B). However, while this is true in absolute terms (5.8 minutes at 27°C vs. 3.7 minutes at 20°C for all individuals; 5.9 minutes at 27°C vs. 2.1 minutes at 20°C for the individuals reared at 20°C), larger relative differences were found in the animals reared at 20°C only (27°C: 20% and 20°C: 21% for all; 27°C: 25% and 20°C: 19% for individuals reared at 20°C). Concomitantly plastic responses are quite similar across selection regimes, with the selection lines showing by 41% (all) and 48% (reared at 20°C) shorter chill-coma recovery times at 20°C compared to 27°C, and the control lines by 40% and 52% shorter ones. We therefore conclude that the extent of the plastic responses in cold tolerance is comparably high across both selection regimes, thus challenging the main hypothesis we wanted to test here.

Effects of different rearing and adult temperatures were strong and persistent, with animals reared or acclimated at the lower temperature showing increased cold tolerance compared to those kept at the higher temperature (cf. Table 1). Similar temperature-induced changes in cold tolerance have been documented in a large variety of insects, suggesting a universal pattern of adaptive phenotypic plasticity [23,50]. In contrast, no consistent effects of adult feeding treatment on cold tolerance were found, despite large variation among inbreeding levels, sexes, and temperature groups. This is in line with our predictions based on earlier results [23], which indicates that cold tolerance is at least not strongly limited by adult food intake. Furthermore, our results revealed no sex differences in cold tolerance as was expected. Likewise, testing for sex differences in temperature stress resistance in an earlier study yielded a non-significant result in 18 out of 22 statistical analyses, suggesting that, in general, both sexes are equally tolerant in *B. anynana*[23]. Except from some group-specific responses, no effects of (earlier) inbreeding on cold tolerance were found.

### Variation in longevity

Longevity was not affected by selection regime, indicating that there is no trade-off between increased cold stress resistance and life span in *B. anynana* (cf. [34]). This notion is further supported by the erratic patterns indicated by the seven interactions in which selection regime was involved in. Studies on *Drosophila* also yielded inconclusive results regarding genetic correlations between cold tolerance and longevity (e.g. [2,15,16,42]). Temperature effects, in contrast, were straight-forward and as predicted. Animals reared at or acclimated to 20°C lived generally longer than animals reared or kept at the higher temperature, thus indicating accelerated rates of ageing at warmer temperatures owing to higher metabolic rates [2,47,51]. Adult food stress had as expected a large impact on longevity, with a lack of food substantially reducing life span [36,37]. Not surprisingly, differences in longevity between acclimation temperature groups were more pronounced in control compared to food-stressed animals, as all latter individuals died within relatively short time spans. Once again, inbreeding yielded no conclusive effects.

Regarding sex differences, females lived longer than males when being reared at 27°C, as has been found in several earlier studies [17,38]. The sex difference was more pronounced in food-stressed than in control animals, which may suggest a re-allocation of resources from reproduction to somatic maintenance under food stress in females. When being reared at 20°C, however, males lived longer than females, but only at the higher adult temperature. This finding may suggest that the respective temperature regime does not only affect longevity as such, but influences sexes in different ways.

## Conclusions

Regarding effects of temperature, feeding treatment, sex, inbreeding and selection regime our results are in good agreement with the hypotheses outlined in Table 1 and thus with earlier findings obtained in *B. anynana* and other insects. However, our principal intention here was to explore plastic responses in populations of different genetic background and thus genotype-environment interactions. Regarding this matter we have indeed detected a number of interactions in which selection regime was involved in, but most of these did not reveal consistent patterns. We have found some evidence though that the lines selected for increased cold tolerance as compared to control lines (1) developed slightly faster at suboptimally low temperatures, (2) showed larger differences in cold tolerance compared to controls when being reared at the lower temperature, (3) and showed more pronounced differences in cold tolerance compared to controls at an adult temperature of 27°C. The former findings may indicate that the lines selected for increased adult cold tolerance may be slightly better equipped to deal with lower temperatures, such that any advantages become exaggerated when experiencing suboptimally low temperatures during development. While thus the plastic response to different rearing temperatures seemed to be somewhat larger in the selection compared to the control lines, plastic responses to different adult temperatures were very similar across selection regimes. Our results thus suggest that plastic capacities were, if anything, only marginally affected by directional selection, and that plasticity was largely independent of the respective trait mean. This suggests that the genetic architecture of the trait mean operates largely independently of the architecture underlying plastic responses. Thus, we found no evidence for an interference of genetic adaptation with plastic capacities (but see [52] for an interspecific comparison).

However, one should keep in mind that our study draws on variation across selection lines, which had been established from a single laboratory source population. While using a single gene pool to experimentally establish genetically differentiated populations has clear advantages, it also involves some limitations which should be acknowledged. Notably, we do not know whether our results are transferable to other populations (and species), and whether they might be affected by the specific population history (e.g. inbreeding, founder effects, laboratory adaptation). Several traits have been shown to readily respond to laboratory conditions, though essentially nothing is known about the effects of laboratory adaptation on plastic responses [53,54]. For logistic reasons such limitations apply to many laboratory experiments. At least some studies indicate that laboratory results may nevertheless have clear relevance for field conditions [54]. In any case our results clearly show that there is not necessarily a link between trait value and the magnitude of plastic responses.

## Competing interests

The authors declare that they have no competing interests.

## Authors' contributions

KFr conducted the experiment, performed the statistical analyses and drafted the manuscript. AD performed the selection experiment. KFi conceived the study, performed the statistical analyses and helped to draft the manuscript. All authors read and approved the final manuscript.
